# Unusual Case of Osseointegrated Dental Implant Migration into Maxillary Sinus Removed 12 Years after Insertion

**DOI:** 10.1155/2017/9634672

**Published:** 2017-03-14

**Authors:** Mauro Laureti, Nicola Ferrigno, Daniele Rosella, Piero Papi, Francesca Mencio, Francesca De Angelis, Giorgio Pompa, Stefano Di Carlo

**Affiliations:** Department of Oral and Maxillofacial Sciences, “Sapienza” University of Rome, Rome, Italy

## Abstract

Displacement of dental implants into the maxillary sinus is not an uncommon event in implant dentistry and may lead to serious complications, such as sinusitis. To avoid systemic problems, performing the removal of the foreign body as soon as possible is suggested. Despite the fact that early implants dislocation has been reported several times, late migration into maxillary sinus has been described by just a few studies. The purpose of this study was to report a rare case of dental implant migration into maxillary sinus after 12 years of function. A 61-year-old woman came to our attention in June 2015 after being visited by an otolaryngologist and being diagnosed with sinusitis and presence of a foreign body into the right maxillary sinus. A panoramic radiograph and a CT scan showed the migration of dental implant sited in 1.6 positions into the maxillary sinus. The implant was removed following a Caldwell-Luc procedure under local anesthesia. Postoperative course was uncomplicated and the patient reported no symptoms of sinusitis after 12 months of follow-up.

## 1. Introduction

Dental implant placement is a high success rate procedure with a great positive impact on patients' quality of life [1, 2]. However, the risk of incurring in short- and long-term complication is sometimes possible. The posterior maxilla is one of the regions where complications are more likely to occur and its rehabilitation may be a demanding challenge for oral surgeons. This area is often characterized by low bone density and quality, fast alveolar ridge reabsorption, maxillary sinus pneumatization, which could lead to a lack of primary stability, and sinus perforation with displacement of dental implants [3]. Use of short implants usually allows implant placement with residual bone vertical height; however, at certain times, it may be necessary to perform sinus floor elevation. To avoid complications, an accurate presurgical planning and surgical experience are needed. Displacement of dental implants into the maxillary sinus is not uncommonly reported in implant dentistry and may lead to serious complications, such as sinusitis, by interrupting mucociliary clearance or producing a tissue reaction [4]. Mostly, there are three ways of treating a dental implant displacement into maxillary sinus: transnasal and transoral endoscopic surgery [5], intraoral approach with anterior-lateral window access to maxillary sinus [6], and no intervention and follow-up only [7]. However, to avoid systemic problems removing the foreign body as soon as possible is suggested [8]. Despite the fact that transsurgical or early implants dislocation has been reported several times, late migration into maxillary sinus has been described just a few times in literature [4, 7, 9–16]. The purpose of this study was to report a rare case of dental implant migration into maxillary sinus after 12 years of function.

## 2. Case Report

**Figure 1:**
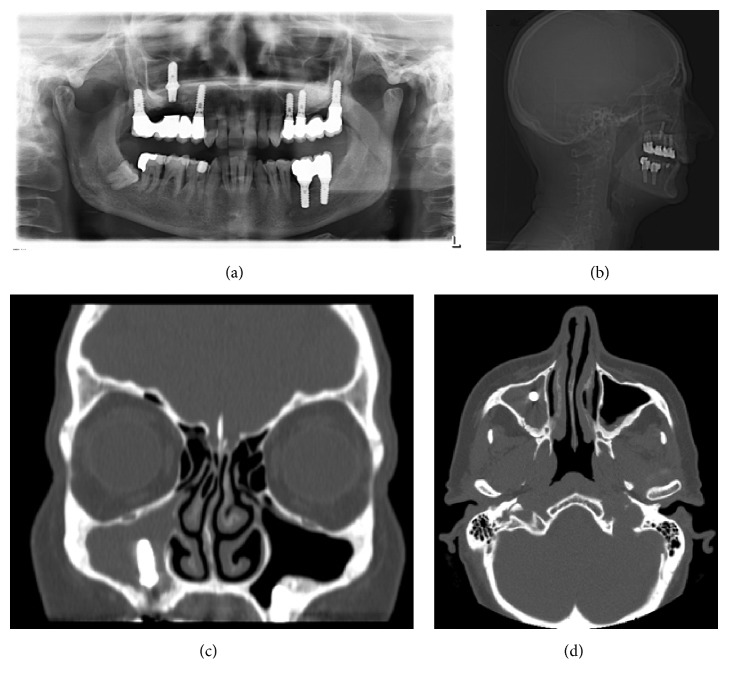
(a) Preoperative panoramic, (b) lateral cephalogram, and (c, d) CT scan radiographies showed the migration of dental implant sited in 1.6 positions into right maxillary sinus and clear signs of sinusitis.

**Figure 2:**
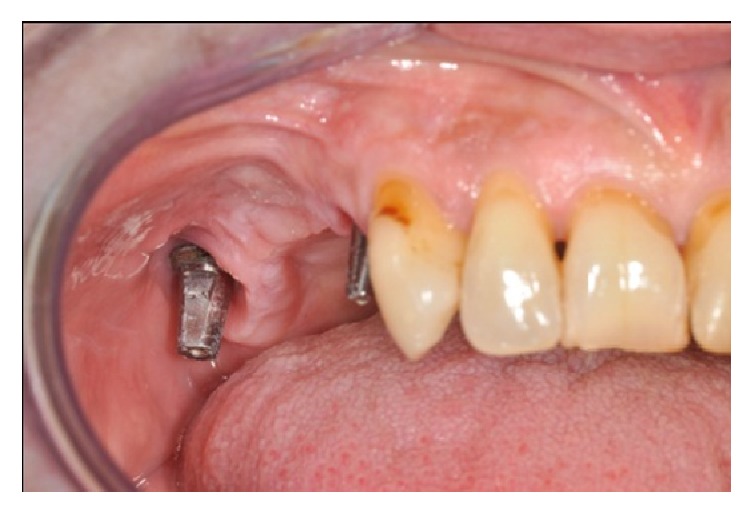
Preoperative clinical view of right posterior maxilla after the prosthesis was removed. The dental implant sited in position 1.6 results missed due to its dislocation into the sinus cavity.

A 61-year-old woman, smoker and being without any uncontrolled systemic diseases, was referred to our department in June 2015 after being visited by an otolaryngologist. A diagnosis of sinusitis and presence of a foreign body into the right maxillary sinus was performed, with no involvement of other paranasal sinuses. A panoramic radiograph and a computed tomography (CT) showed the migration of dental implant sited in 1.6 position into the right maxillary sinus (Figure 1). The implant-supported prosthesis had no sign of mobility and the oral mucosa appeared to be normal with no evidence of oroantral fistula (Figure 2).

According to medical records, to replace her missing teeth in the posterior right maxilla, a sinus lift surgery with simultaneous implants placement in April 2003 was performed. Three tissue level dental implants with rough surfaces (Soft Tissue level Standard Plus, Institut Straumann AG, Basel, Switzerland) were inserted: site 1.4: ⌀4.1 × 12 mm; site 1.6: ⌀4.8 × 10 mm; site 1.7: ⌀4.8 × 10 mm. The procedure was completed with a lateral window approach using native bone mixed with a xenograft derived from bovine bone mineral (BioOss, Geistlich, Switzerland) and a porcine collagen membrane (BioGide, Geistlich, Switzerland) was placed to protect lateral window of the sinus cavity. After 6 months of osseointegration, a provisional implant-supported fixed prosthesis was delivered to patient and after another 3 months the final restoration made of gold-platinum ceramic alloy was completed. The patient was regularly monitored for the first years after surgery and occasionally observed during last decade. Irregular appointments with a dental hygienist were scheduled. No specific treatment for peri-implantitis was completed. Radiographs showed a progressive peri-implant bone loss and peri-implantitis progression, which led to implant migration into the sinus cavity.

**Figure 3:**
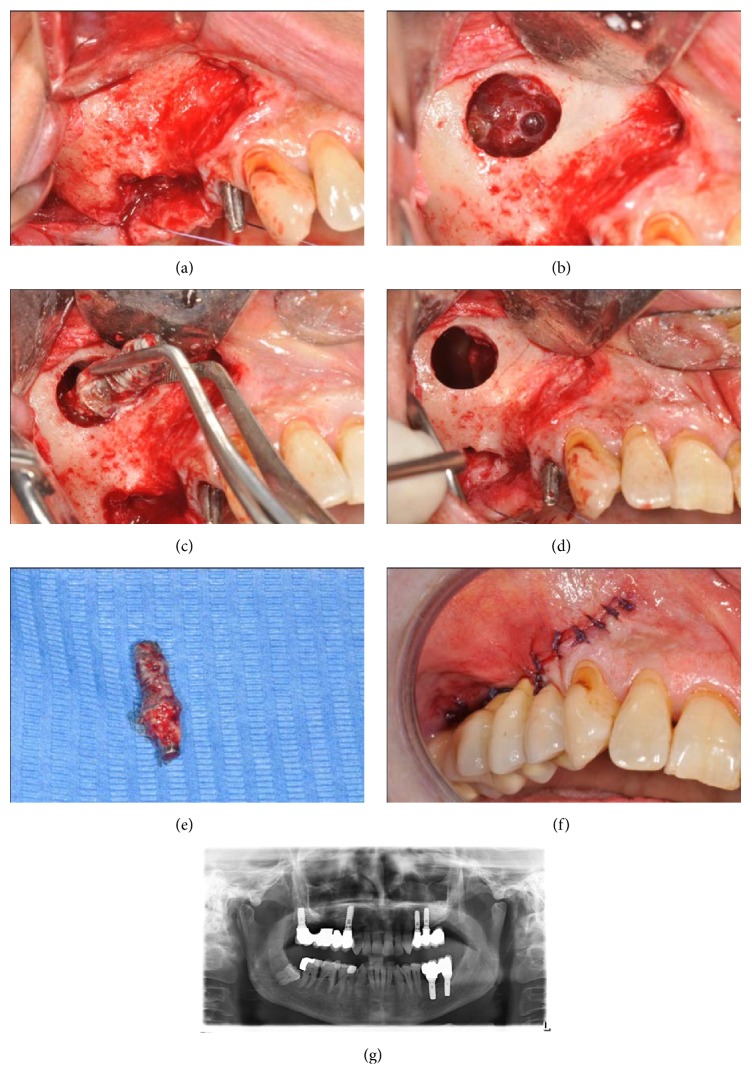
(a) Clinical situation after the flap was raised. (b) Photograph of the right maxillary sinus after a lateral window access showing sinusitis secondary to implant displacement. (c) Picture of the implant removal with forceps. (d) Maxillary sinus situation after its surgical revision. (e) Extra-oral view of the removed implant. (f) Clinical situation after the flap was sutured and the fixed prosthesis was cemented. (g) Radiographic view of the right maxillary sinus soon after the implant was removed.

**Figure 4:**
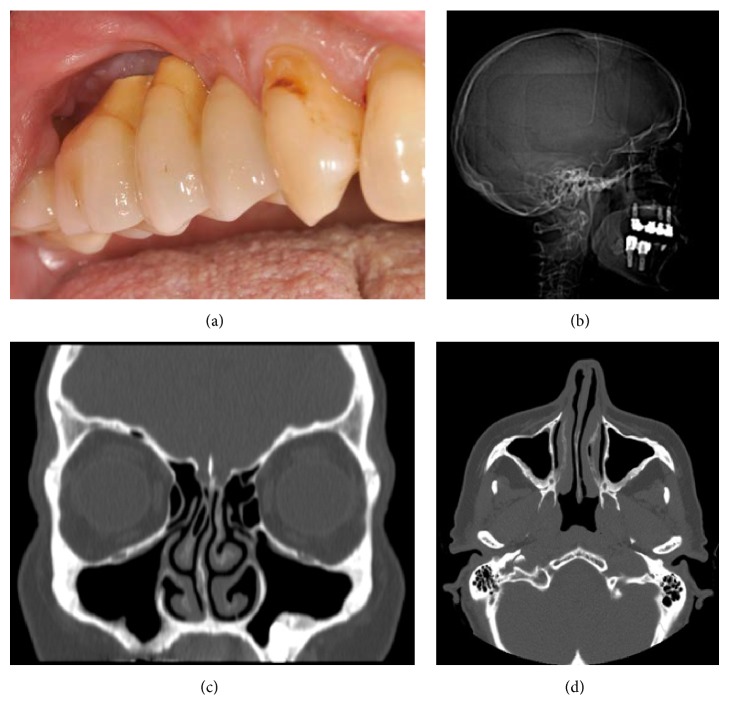
(a) Clinical view of the healed surgical area. (b) Lateral cephalogram without any sign of the foreign body into the sinus. (c, d) The CT scans show normal mucosal thickness and no opacification of the right maxillary sinus.

The implant was removed following a Caldwell-Luc (CL) procedure in July 2015. Patient was operated under local anesthesia with a later window access of the maxillary sinus. The buccal aspect of the flap was raised to approach anterior bony wall of maxillary sinus. A straight hand piece and a round burr were used to complete osteotomy and gain access into the cavity. The Schneiderian membrane was eliminated and the maxillary sinus was fully revisited after implant removal. After irrigation with sterile saline solution, flap was closed by primary intention with simple interrupted suture (Figure 3). Antibiotic therapy (Amoxicillin and Clavulanic acid 875 mg/125 mg; Augmentin, GSK, UK) and nonsteroidal anti-inflammatory drugs (Ibuprofen 600 mg, Brufen, Abbott, USA) were prescribed for 7 days after surgery. Chlorhexidine mouthwash (Corsodyl, GSK, UK) was administered for 2 weeks until suture removal. Postoperative course was uncomplicated and patient reported no symptoms of sinusitis after 12 months of follow-up, as confirmed by CT scans (Figure 4).

## 3. Discussion

Poor bone quality and quantity and proximity to anatomical structures, such as maxillary sinus, make the posterior maxilla one of the areas at greater risk of complications. Soft bone and/or overpreparation of implant site may result in lower primary stability, which may induce fibrous encapsulation instead of osseointegration with higher implant failure rates [17]. Alveolar ridge reabsorption and progressive pneumatization of sinus cavity result in a reduced bone height of the posterior maxilla, resulting as a primary cause of implant displacement in the maxillary sinus [11]. Presurgical CT/CBCT evaluation is helpful for studying residual alveolar bone quality and quantity, revealing anatomical landmarks, and facilitating diagnosis of possible silent sinus pathology. Computer-guided surgery as well as the use of surgical splints may also be helpful, especially for less experienced implantologists [16]. Errors of surgical planning may result in implant placement into areas with poor bone quality and quantity, resulting in sinus perforation and implant dislocation. In case of inadequate residual bone height, alternative treatment strategies should be considered, such as short implants, tilted implants, or bone graft procedures [18]. Regev et al. [19] reported in 1995 the first case of implant dislocation; then, a growing number of case reports or larger series have been described in literature over the last two decades [4–16, 19–22]. The majority of dental implants are displaced immediately or soon after implantation: as described in a recent study of Jeong et al. [20], in the articles reviewed, only 7/49 dental implants were dislocated 1 year after placement or after loading. While reasons of early implant dislocation have been described, mechanisms behind migration of dental implants into the maxillary sinus several years after osseointegration are harder to understand. Galindo-Moreno et al. [21] suggested a higher incidence of implant migration into maxillary sinus for cylindrical and narrower implants as compared to conical and wider ones. It is probably related to better primary stability for conical and bigger-diameter implants. Similar findings have been reported by Sgaramella et al. [16]: displacement was more frequent for cylindrical implant (62.5%) but no correlation was found between implant diameter and dislocation. There is evidence that close proximity between osseointegrated implants and maxillary sinus could lead to complications [7]. Local infection of peri-implant tissue [23, 24] may allow its spread from oral cavity to maxillary sinus. Peri-implantitis evolution could lead to further complications as implant migration into sinus cavity. The reasons of implant migration several years after osseointegration are still unknown. Three possible mechanisms to explain late implant displacement have been proposed: inflammatory reaction to the implant secondary to infection, bone reabsorption caused by incorrect distribution of occlusal forces, and changes in intrasinal and nasal pressures producing a suction effect [18]. The first one was probably the cause of implant migration in our case report. Whatever the cause, dislocated implant should be removed from maxillary sinus as soon as possible to avoid further complications [8]. Removal of a foreign body into the sinus cavity may be performed using different techniques: extraction through the intraoral fistula, direct approach by opening a lateral window into the sinus, and transnasal or transoral endoscopic surgery [22]. While the majority of dislocation cases reported in literature were treated with a CL procedure, transnasal approach with functional endoscopic sinus surgery (FESS) has proven to be less aggressive and it allows an endoscopic control and treatment of the maxillary antrum and other paranasal sinuses, which can be secondarily involved by infections starting from the maxillary cavity [8]. In the event of an oroantral fistula or when the foreign body has a considerable size, a direct approach may be needed. In the present case, due to poor current economic condition of the patient and long waiting list to undergo a FESS procedure in our public hospital, a CL approach was preferred. Furthermore, a direct procedure allowed a better visualization of the surgical area removing the big size foreign body (6.5 mm of neck diameter × 10 mm in length). It was also performed under local anesthesia with no need to hospitalization. After 12 months of follow-up, no clinical signs of sinusitis have been reported. A CT scan showed a maxillary sinus without opacification, suggesting a normal patency of cavity.

## 4. Conclusion

Late migration of dental implants after their osseointegration is a very rare event and reasons still remain unknown. Good primary stability and sufficient bone quality and quantity at implant insertion are important factors to prevent implant displacement. To avoid further complications, implants migrated into maxillary sinus should be removed immediately. A Caldwell-Luc approach is a simple procedure, which could be performed in direct view and local anesthesia by oral and maxillofacial surgeons. In case of sinusitis, patient should also be referred to an otorhinolaryngologist for further analysis.
